# TFmiR: a web server for constructing and analyzing disease-specific transcription factor and miRNA co-regulatory networks

**DOI:** 10.1093/nar/gkv418

**Published:** 2015-05-05

**Authors:** Mohamed Hamed, Christian Spaniol, Maryam Nazarieh, Volkhard Helms

**Affiliations:** Center for Bioinformatics, Saarland University, 66041 Saarbrucken, Germany

## Abstract

TFmiR is a freely available web server for deep and integrative analysis of combinatorial regulatory interactions between transcription factors, microRNAs and target genes that are involved in disease pathogenesis. Since the inner workings of cells rely on the correct functioning of an enormously complex system of activating and repressing interactions that can be perturbed in many ways, TFmiR helps to better elucidate cellular mechanisms at the molecular level from a network perspective. The provided topological and functional analyses promote TFmiR as a reliable systems biology tool for researchers across the life science communities. TFmiR web server is accessible through the following URL: http://service.bioinformatik.uni-saarland.de/tfmir.

## INTRODUCTION

Among many genetic regulators, transcription factors (TFs) and microRNAs (miRNAs) are the essential key players for regulating gene expression (1). Together they play important roles in regulating virtually all cellular processes such as differentiation, proliferation, survival and apoptosis (2). Also genetic disorders and complex diseases including cancer are mostly associated with perturbations of the interwoven regulatory circuit between TFs and miRNAs (3,4). TFs and miRNAs frequently form Feed Forward Loops (FFLs) and other network motifs to regulate cellular transcription in a connective manner (4,5). Therefore, utilizing the combined regulatory information on TFs and miRNAs as well as their target genes could shed light on key driver genes and miRNAs in human diseases and, in turn, suggests novel therapeutic strategies in disease treatment (5,6).

Several databases have been developed in order to facilitate research on transcriptional and post-transcriptional interaction types between TFs, miRNAs and target genes. For instance, TransFac (7), OregAnno (8) and MsigDB (9) provide compilations of TFs regulating genes (*TF* → *gene*). TransmiR (10) provides information on which TFs regulate miRNAs (*TF* → *miRNA*). mirTarBase (11), TarBase (12) and miRecords (13) collect target genes of miRNAs (*miRNA* → *genes*) in different organisms. Although still little is known about miRNA-mediated miRNA regulations, recent studies reported plausible evidences that miRNAs may regulate the expression of other miRNAs as well as their target genes (5,14). Thus, *miRNA* → *miRNA* interactions were computationally predicted and made available in the PmmR database (15).

Despite the general availability of such databases, generalized repositories integrating different kinds of molecular interactions and enabling to analyze their contributions to diseases are still missing. To this end, we present TFmiR, a web server that allows for integrative and comprehensive analysis of interactions between a set of deregulated TFs/genes and a set of deregulated miRNAs within the relevant pathways of a certain disease. The tool unravels the disease-specific co-regulatory network between TFs and miRNAs and performs over representation analysis (ORA) for the involved TFs/genes and miRNAs. Our web server also detects FFLs consisting of miRNAs, TFs and co-targeted genes (TF–miRNA co-regulatory motifs) and statistically assesses the functional homogeneity between the co-regulated targets. Furthermore, TFmiR utilizes seven different methods for identifying key network players that could possibly drive oncogenic processes of diseases and thus could act as potential drug targets. Especially when combined with experimental validation, these putative key players as well as the novel TF–miRNA co-regulatory motifs could promote novel insights to develop new therapeutic approaches for human diseases.

## MATERIALS AND METHODS

### Description

**Figure 1.**
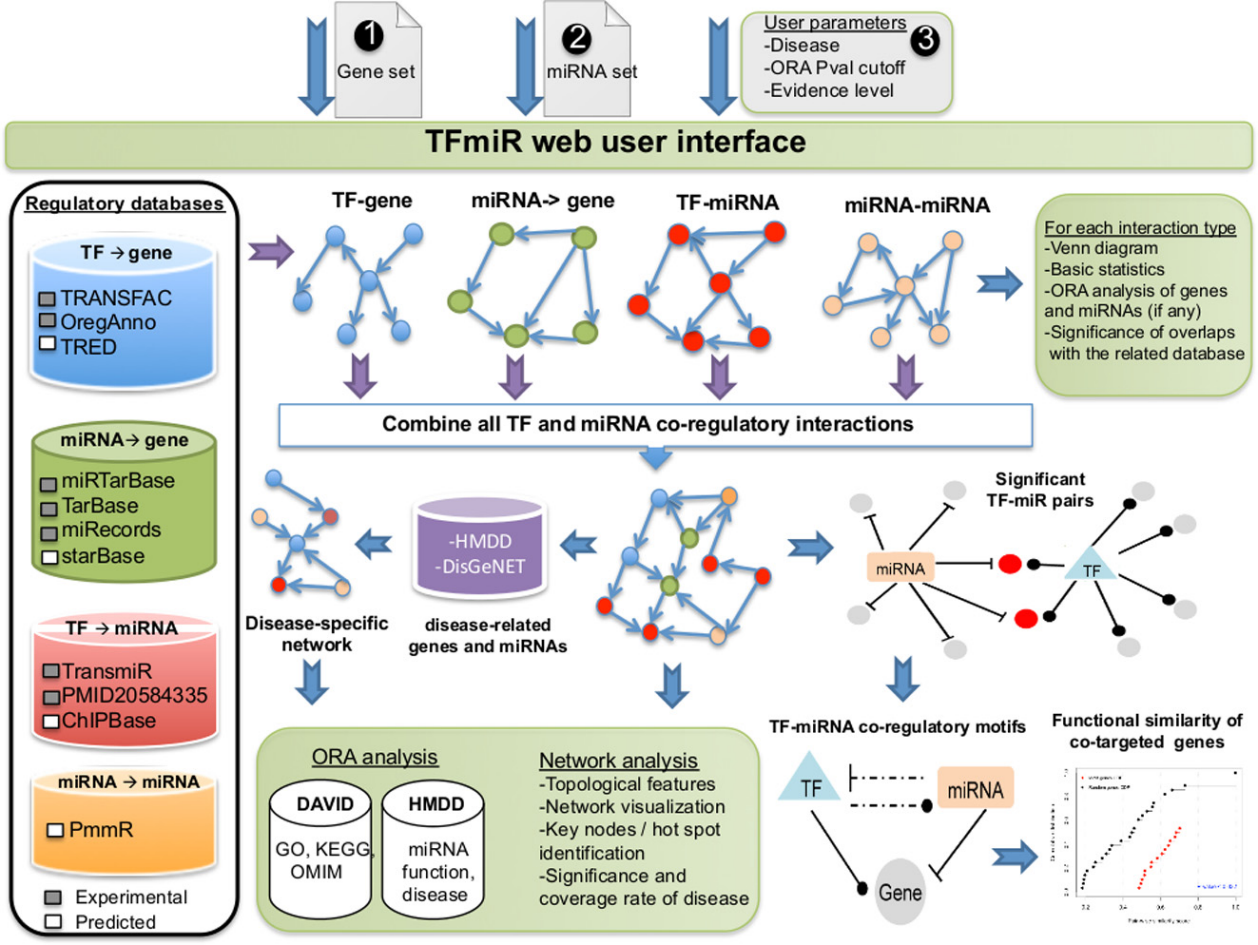
A system level overview of the TFmiR architecture describing the incorporated databases, data flows and output downstream analysis.

TFmiR integrates genome-wide transcriptional and post-transcriptional regulatory interactions to elucidate human diseases. For a specified disease and based on user-supplied lists of deregulated genes/TFs and miRNAs, TFmiR investigates four different types of interactions, *TF* → *gene, TF* → *miRNA, miRNA* → *miRNA* and *miRNA* → *gene*. It also unravels the circuitry between miRNAs, TFs and target genes with respect to specified diseases. For each interaction type, TFmiR utilizes information provided by established and curated regulatory databases of both predicted and experimentally validated interactions (Figure 1) whereby all duplicate interactions were removed. For *TF* → *miRNA* interactions, we also integrated manually curated regulatory relationships from (∼5000) published papers (16). From the predicted *miRNA* → *miRNA* interactions in the PmmR database (15), we considered only the best hits having *score* <0.2, which was computed as the normalized path length between the two involved miRNAs. The incorporated predicted *miRNA* → *gene* interactions were retrieved from starBase (17) by selecting only those predictions confirmed by three out of five prediction algorithms (targetScan (18), picTar (19), RNA22 (20), PITA (21) and miRanda (22)). Supplementary Table S1 lists the included databases and the number of regulations available for each interaction type. In total, TFmiR currently integrates information on almost 10 000 genes, 1856 miRNAs, ∼3000 diseases including subtypes and more than 111 000 interactions.

### TFmiR user input scenarios

TFmiR can be called through two scenarios. If the user submits two RNA sets (a set of deregulated mRNAs/genes and a set of deregulated miRNAs), the tool will return regulatory interactions based on the provided deregulated genes and deregulated miRNAs. In the second scenario, a user submits only a set of deregulated genes. In this case, TFmiR identifies the set of miRNAs whose target genes as well as regulator TFs are significantly enriched within the input deregulated genes using the hypergeometric distribution function followed by the Benjamini–Hochberg (BH) adjustment with a cutoff value of 0.001. Sample input files of the deregulated genes and miRNAs are provided in Supplementary Figures S1 and S2. The user can optionally set the *P*-value cutoff (default is 0.05) for ORA on the resulting network nodes (genes/miRNAs). Finally, the user can control the evidence level (experimentally validated, predicted or both) for the constructed regulatory interactions that will be used in the subsequent network analysis.

### Functionality of TFmiR

TFmiR pools all four interactions types based on the input deregulated genes and miRNAs and accordingly generates the entire combinatorial regulatory network. If a disease was selected, TFmiR uses data retrieved from the human miRNA disease database (HMDD) (23) as well as DisGeNET (a database for gene-disease association) (24) as sources for disease-associated miRNAs and genes, respectively. Interactions whose target nodes or regulator nodes are known to be associated with the disease compose the putative disease-specific network. TFmiR then offers three levels of downstream analysis: (i) the regulatory subnetwork of the four interaction types, (ii) the combined network of all interaction types and (iii) the disease-specific network (if disease was selected).

For each interaction type subnetwork of *regulator* → *target* links, we display the total number of targets and regulators in the corresponding interaction databases, a Venn diagram depicting the overlap between the input deregulated targets (miRNAs/genes) and the targets of the input deregulated regulators (genes/miRNAs) available from the database. The significance of overlap is computed using the hypergeometric distribution test as an indicator how representative the provided deregulated inputs are for the known cellular targets of the considered regulators. This measure suggests whether the follow-up downstream analysis is likely to yield meaningful functional enrichments or disease associations. To avoid the effect of false-positives in the *regulator* → *target* databases and to account for a different number of targets for the input deregulated regulators, a randomization test is conducted (*n* = 1000). Furthermore, TFmiR carries out ORA for both gene analyses and miRNA sets comprising the interaction subnetwork.

For gene set analysis, TFmiR employs DAVID (25) to check for enrichment of GO terms (BP subcategory), KEGG pathways and OMIM diseases as well as for clustering the genes based on their functional similarities. For miRNA set analysis, we used the miRNA-functional association data and miRNA-disease association data from HMDD to statistically relate the functional and disease terms to the miRNA set.

For levels 2 and 3, TFmiR calculates for each network basic topological features, relevance to the disease-associated genes/miRNAs by testing the overlap significance with the network nodes, degree distribution plot, ORA analyses for both gene and miRNA nodes, network key nodes, and detects 3-node motifs. To measure the strength of correlation between the potential disease-specific network, the input disease and the input deregulated genes and miRNAs, we compute a coverage ratio (CR) between the nodes of the disease-specific network and the nodes of the entire combined network.

\begin{equation*} C_{R} = \frac{N_{d}}{N_{t}} \end{equation*}

Here, *N*_*d*_ represents the number of disease-specific network nodes and *N*_*t*_ represents the total number of nodes in the entire network. We also calculate the CR ratio between the edges of the two networks. All resulting networks are visualized using the interactive Cytoscape-web viewer (26).

### Identification of network key nodes

We defined the key nodes as the top 10% highest centrality nodes of the TFs, miRNAs and genes in the disease-specific and whole network. TFmir uses degree centrality, closeness centrality, betweenness centrality and eigenvector centrality as well as the common and union sets of the key nodes identified by these four measures. We also determine the minimal set of dominating nodes that regulate the entire network. This can be modeled as the following optimization problem: Let graph *G(V,E)* be a connected graph, *n* = |*V*|, adj is the adjacency matrix of G, and *adj*(*i, i*) = 0, *X* is a binary array of size *n*, such that *X*(*i*) = 1 if node *i* was marked as a key node, and 0 otherwise. Then the objective function is:

\begin{eqnarray*} \text{min}& & \sum _{i = 1}^{n} X(i) \\ \text{subject to } \forall i & & \sum _{i }^{n} adj(i,j).X(j) >= 1. \end{eqnarray*}

The last constraint guarantees that every node in the network must have at least one key node in its neighborhood. To solve such an optimization problem, we apply the algorithm presented by Rai *et al*. 2009 (27) to search for the minimum dominating node set.

### Identification of TF–miRNA co-regulatory motifs

**Figure 2.**
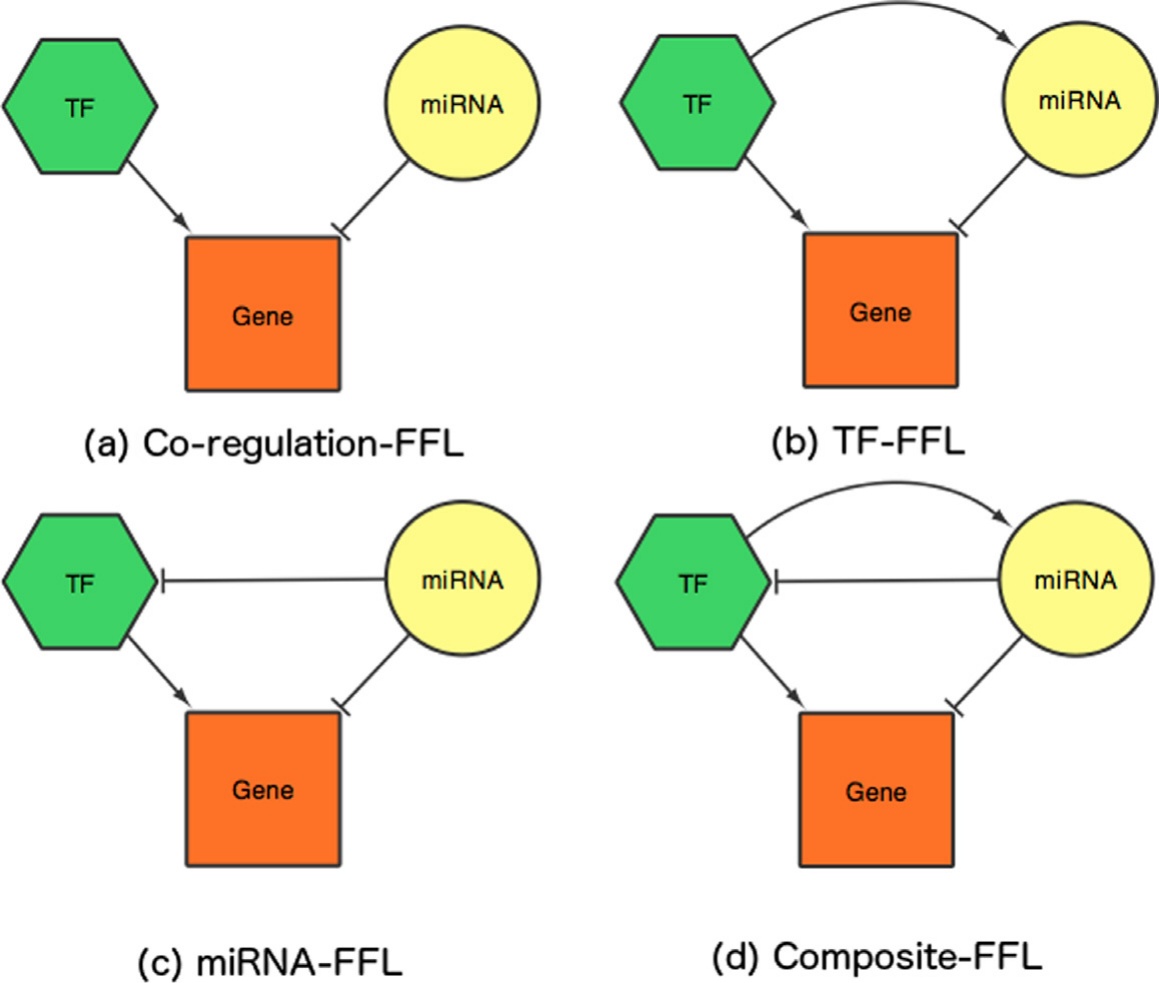
Schematic illustration of the four motif types detected in TFmiR. All motifs contain a TF, a miRNA and a common target gene.

FFLs are interconnection patterns that recur in many different parts of a network and form key functional modules (5,28). They have been demonstrated as one of the most important motif patterns in transcriptional regulation networks (28) that govern many aspects of normal cell functions and diseases (29,30). Here, TFmiR identifies four types of 3-nodes motifs (three FFLs and one co-regulation motif) consisting of a TF, a miRNA and their co-targeted gene and defines them as TF–miRNA co-regulatory motifs (Figure 2). (i) The Coregulation-FFL includes only TF regulation of a target gene as well as miRNA repression of that target gene. (ii) The TF-FFL includes TF regulation of the expression of both a miRNA and a target gene and it also includes miRNA repression of that target gene. (iii) The miRNA-FFL includes miRNA repression of both a TF and a target gene, as well as TF regulation of this target gene. (iv) The Composite-FFL describes TF regulation of both a miRNA and a target gene as well as miRNA suppression of that TF and that target gene.

#### Identifying significant TF–miRNA co-occuring pairs

We identified statistically significant TF and miRNA pairs that cooperatively regulate the same target gene using the hypergeometric distribution and evaluated *P-values*:

\begin{equation*} P\rm {-value} = 1 - \sum _{i = 0}^{x}\frac{{k \atopwithdelims ()i}{M-k \atopwithdelims ()N-i}}{{M\atopwithdelims ()N}} \end{equation*}

where *k* is the number of target genes of a certain miRNA, *N* is the number of genes regulated by a certain TF, *x* is the number of common target genes between these TF and miRNA, and *M* is the number of genes in the union of all human genes targeted by human miRNAs and all human genes regulated by all human TFs in our databases. Then, multiple test correction was done by determining the false discovery rate according to BH (31) method and only those pairs with a adjusted *P*-value <0.05 were selected as significant TF–miRNA pairs.

#### Construction of candidate TF–miRNA-gene FFLs

All interactions associated with the significant TF–miRNA pairs are represented as connectivity matrix, *M*, such that *M*_*ij*_ = 1 if regulator *i* regulates target *j* where *i* ∈ (*TF, miRNA*) and *j* ∈ (*TF, miRNA, gene*). Then, we scan all the 3*3 submatrices of *M* that represent each type of the four considered FFL topologies (Figure 2).

#### Significance of the FFL motifs

To evaluate the significance of each FFL motif type, we compare how often they appear in the real network to the number of times they appear in randomized ensembles preserving the same node degrees. In order to retain stronger attachment of biological key driver nodes, we applied a degree preserving randomization algorithm of the ‘*igraph*’ R-package. For 2 × *L* steps, two edges *e*_1_ = (v_1_, v_2_) and *e*_2_ = (v_3_, v_4_) are randomly chosen from the network and rewired such that the start and end nodes are swapped, i.e. *e*_3_ = (v_1_, v_4_) and *e*_4_ = (v_3_, v_2_) if $\lbrace e_3,e_4\rbrace \not\in V$. The random networks were constructed 100 times and compared to the real network. The *P-value* is calculated as

\begin{equation*} P\rm {-value} = \frac{N_{h}}{N_{r}} \end{equation*}

where *N*_*h*_ is the number of random times that a certain motif type is acquired more than or equal to its number in the real network, and *N*_*r*_ is 100. We also calculate the Z score for each motif type to examine by how many standard deviations the observed real motif was above or below the mean of the random ones.

\begin{equation*} \rm {Zscore} = \frac{N_{o}-N_{m}}{\sigma } \end{equation*}

Here *N*_*o*_ is the number of motifs observed in the real network, whereas *N*_*m*_, and σ are the mean and standard deviation of the motif occurrence in 100 random networks, respectively.

### Functional homogeneity

In order to evaluate the biological evidence of the identified TF–miRNA co-regulatory motifs and better understand their functional roles, TFmiR allows the user to analyze the GO semantic similarity for all pairs of genes targeted by the same TF and miRNA pair or for all pairs of genes regulated by the TF or the miRNAs of that TF–miRNA pair (see Supplementary Figure S3). The GoSemSim R package (32) is used to compute the semantic similarity scores according to the Gene Ontology (GO) annotations. Statistical significance is determined by randomly selecting the same number of genes (co-targeted genes or co-regulated genes) from all Entrez genes with GO annotations, and computing their similarity scores. The permutation procedure is repeated 1000 times. Then, we carry out a Kolmogorov–Smirnov test to check whether the functional similarity scores of all gene pairs from the FFL motif are significantly higher than that of randomly selected pairs.

## RESULTS

### Case study

TFmiR was applied to several data sets related to complex diseases such as cancer, Alzheimer and diabetes. In a recent study on breast cancer (6), we identified 1262 deregulated genes and 121 deregulated miRNAs using gene and miRNA expression data from the TCGA portal (https://tcga-data.nci.nih.gov/tcga/). These two sets of deregulated genes and miRNAs are the default sample input files provided by the TFmiR web server. Next, TFmiR was used to reveal the co-regulation network between the deregulated genes/TFs and deregulated miRNAs and to better understand the pathogenic mechanisms associated with breast tumorigenesis. As user input parameters we set the *P-value* cutoff to 0.05, disease was set to breast neoplasms, and the evidence level was set to both experimentally validated and predicted interactions.

For this data set TFmiR constructed a total of 427 regulatory interactions comprising 263 nodes of deregulated miRNAs and deregulated TFs/genes. The breast cancer-specific network involved 345 interactions and 212 nodes of deregulated miRNAs and genes with node and edge CR of 80.6% and 80.8%, respectively. The provided ORA analysis of the disease network nodes revealed their implications in many cancer types as well as cancer-related KEGG pathways. Moreover, ORA analysis of the network miRNAs showed their involvement in cancerogenesis of multiple organs such as lung neoplasms, ovarian cancer and adenocarcinoma (Supplementary Table S2). Additionally, TFmiR identified 22 key network players (10 genes and 12 miRNAs) based on the union set of four centrality measures described above (Supplementary Table S3). Interestingly, some of the identified key genes such as BRCA2, ESR1, AKT1 and TP53 were previously implicated and significantly mutated in breast cancer samples (33). More importantly, the protein products of the genes ESR1, TP53, TGFB1, AKT1 and BRCA2 are binding targets for anti-breast cancer drugs (6) (Supplementary Table S4).

Next, we examined the TF–miRNA co-regulatory motifs that were significantly enriched in the entire interaction network. We identified 53 FFL motifs (three composite-FFLs, two TF-FFLs, six miRNA-FFLs and forty-two coreg-FFLs). An interesting motif involving the TF SPI1, the miRNA hsa-mir-155 and the target gene FLI1 reveals how FFL motifs may help to better understanding the pathogenicity of breast cancer (Supplementary Figure S4 from the tool). Recent studies reported that the oncogene SPI1 is involved in tumor progression and metastasis (34,35). However, the co-regulation of the oncogene FLI1 (36) by both SPI1 and the oncomiR hsa-mir-155 was not reported before. As the co-regulated genes of SPI1 and hsa-mir-155 have significantly more similar cellular functions than randomly selected genes (Supplementary Figure S5), this FFL motif provides novel insights into SPI1-miRNA networks alteration in breast cancer and suggests a cooperative functional role between SPI1 and potential miRNA partners.

## DISCUSSION

Compared to the web services of related databases and tools such as Transmir (10), ChIPBase (37), CircuitsDB (38), starBase (17), miR2Disease (39) and cGRNB (40), our TFmiR web server has several distinctive features: (i) TFmiR performs integrative analysis of molecular interactions between a set of deregulated genes and a set of deregulated miRNAs within or without the pathogenic pathways of a certain disease. In contrast, the above mentioned web tools only search the regulatory interactions of a single gene or a single miRNA. (ii) TFmiR performs a rich network analysis, TF–miRNA co-regulatory motif detection, network visualization, statistical significance of the extracted interactions, and ORA analysis for each interaction type, the combined interaction network, and the disease network. Such an integrated analysis is not provided by other web tools. (iii) TFmiR allows the user to retrieve either experimentally validated or predicted interactions or both. Such an option is not available using the other tools. In a somehow similar fashion, DisTMGneT (41) was developed for obtaining cancer-specific network based on user-selected sets of deregulated genes and miRNAs. However, it lacks the downstream analysis, the varieties of user input parameters, and it is limited to a predefined set of miRNAs and genes as well as cancer disease. Also miRTrail (42) performs ORA and Gene Set Enrichment analyses of interactions of genes and miRNAs based on expression profiles. However, it explores only *miRNA* → *gene* interactions.

## CONCLUSION

We developed TFmiR as a comprehensive web server for integrative analysis of the molecular interactions between TFs/genes and miRNAs and their interwoven critical roles in the pathology of human diseases. TFmiR provides an extended downstream analysis, a variety of user parameters, user input scenarios and incorporates information from various well-established regulatory databases. TFmiR is based on user-provided sets of deregulated genes and/or miRNAs regardless of the data producing technologies of either microarray experiments, next generation sequencing or PCR. We showed that unlike the traditional separate analysis of gene expression profiles (43,44) or the aberration of miRNA expression in cancer tissues (45,46), this integrated molecular analysis of deregulated miRNAs and genes using TFmiR was able to uncover literature confirmed core regulators as well as important new aspects of the TF/gene-miRNA interactomes, their co-regulation mechanisms, and the underlying pathogenesis of human breast cancer. The novel hub nodes of TFs/miRNAs could be further experimentally investigated as new potential drug targets. TFmiR was also able to characterize important TF miRNA co-regulatory motifs whose co-regulated genes form cooperative functional modules in breast oncogenesis processes.

## OUTLOOK AND PERSPECTIVE

TFmiR is planned to be integrated with other useful ORA tools such as KeyPathwayMiner (47), GiGA (48), HotNet (49) and jActiveModules (50) to allow the user to benefit their advances within TFmiR. We also intend to allow for submitting multi case expression data and times series data as well as the currently supported case/control data. Finally, expanding the TFmiR to elucidate the regulatory mechanisms of cellular processes (ex. stem cell differentiation) in addition to diseases would sort TFmiR of great interest for wide range of researchers and most of life science community.

## SUPPLEMENTARY DATA

Supplementary Data are available at NAR Online.

SUPPLEMENTARY DATA
