# Supplementary material for: TFmiR: a web server for constructing and analyzing disease-specific transcription factor and miRNA co-regulatory networks
Source: Nucleic Acids Res. 2015 May 5;43(Web Server issue):W283–8. doi: 10.1093/nar/gkv418 (PMC4489273; doi:10.1093/nar/gkv418)
Supplement: SUPPLEMENTARY DATA [file supp_43_W1_W283__index.html]

TFmiR: a web server for constructing and analyzing disease-specific transcription factor and miRNA co-regulatory networks — TFmiR: a web server for constructing and analyzing disease-specific transcription factor and miRNA co-regulatory networks — SUPPLEMENTARY DATA 

# TFmiR: a web server for constructing and analyzing disease-specific transcription factor and miRNA co-regulatory networks

## SUPPLEMENTARY DATA

**Files in this Data Supplement:**

- SUPPLEMENTARY DATA
