# Supplementary material for: TFmiR: a web server for constructing and analyzing disease-specific transcription factor and miRNA co-regulatory networks
Source: Nucleic Acids Res. 2015 May 5;43(Web Server issue):W283–8. doi: 10.1093/nar/gkv418 (PMC4489273; doi:10.1093/nar/gkv418)
Supplement: SUPPLEMENTARY DATA [file supp_gkv418_nar-00605-web-b-2015-File008.pdf]

Supplementary Materials of TFmiR web server

Mohamed Hamed[1], Christian Spaniol[1], Maryam Nazarieh[1], and Volkhard Helms[1]*

Supplementary tables

Table S1. The integrated databases and interaction types in TFmiR.

Interaction	Databases (P/E) *	Genes	miRNAs	Regulatory links	Version /frozen date
**TF→gene**	TRANSFAC (E) (1)	1279	--	2943	V11.4
	OregAnno (E)(2)	1132	--	1083	Nov 2010
	TRED (P) (3)	3038	--	6462	2007
**TF→miRNA**	TransmiR (E) (4)	158	175	567	V1.2, Jan 2013
	PMID20584335 (E) (5)	58	56	102	Apr 2009
	ChipBase (P) (6)	119	1380	33087	V1.1, Nov 2012
**miRNA → gene**	miRTarBase (E)(7)	2244	551	5640	V4.5, Nov 2013
	TarBase (E) (8)	422	79	492	V7.0
	miRecords (E)(9)	543	157	780	Mar 2009
	starBase (P)(10)	5720	249	56051	V2.0, Sept 2013
**miRNA→miRNA**	PmmR (P) (11)	--	312	3846	Mar 2011

* (P) means predicted interactions and (E) means experimentally validated interactions.

Table S2. The most significant functions and diseases enriched in the miRNA nodes of the breast cancer disease network (12).

Category	Term	miRNAs Count	P-value
**Function**	Epithelial-mesenchymal transition	17	0.022
**Function**	glucose metabolism	4	0.048
**Disease**	Breast Neoplasms	67	1.43E-25
**Disease**	Lung Neoplasms	50	4.33E-17
**Disease**	Neoplasms	44	3.15E-15
**Disease**	Ovarian Neoplasms	43	1.30E-14
**Disease**	Adenocarcinoma	27	2.59E-13
**Disease**	Pancreatic Neoplasms	39	7.30E-13
**Disease**	Prostatic Neoplasms	41	3.49E-12
**Disease**	Melanoma	45	1.25E-11
**Disease**	Colonic Neoplasms	32	4.67E-11
**Disease**	Colorectal Neoplasms	45	5.69E-11

© Oxford University Press 2005

Table S3. Key genes and miRNAs in the breast cancer network (12).

**Key genes**	E2F6, TP53, SPI1, TGFB1, SMAD4, ESR1, TERT, E2F3, BRCA2, AKT1
**Key miRNAs**	hsa-mir-148a, hsa-mir-21, hsa-mir-93, hsa-mir-152, hsa-mir-106b, hsa-mir-143, hsa-mir-200c, hsa-mir-27a, hsa-mir-23a, hsa-mir-22, , hsa-mir-146a, hsa-mir-335

Table S4. The identified key gene nodes in the breast cancer network (12) whose protein products are targeted by anti-cancer drugs. (1) means that at least one drug that targets this gene product is reported in this database, and (0) means no drugs are reported for the respective gene in this database. Not included are substances that are known to be cancerogenous or mutagenic.

Target gene	Drug and antineoplastic agents	CTD	PharmGKB	Cancer Resource
**AKT1**	U 0126;tyrphostin AG 1478; Ursodeoxycholic Acid;Valproic Acid;tyrphostin AG 1024; trametinib; Tretinoin	1	0	1
**BRCA2**	Tretinoin; trichostatin A; Estradiol; transplatin; troglitazone; Tunicamycin; fulvestrant	1	0	1
**ESR1**	exemestane;tamoxifen	0	1	1
**TGFB1**	Doxorubicin; Fluorouracil; Thalidomide; Entinostat; Hyaluronidase	0	0	1
**TP53**	4-biphenylmine; alliin; Apigenin; Atropine;bicalutamide;butylidenephthalide	0	0	1

Supplementary Figures

genes.bc.diff.txt

```
AATK      -1
ABCB8     1
ABCG4     1
ABHD10    1
ABLIM1    -1
ABT1      1
ABTB1     -1
ACAN      1
ACBD3     1
ACCN2     1
ACOT2     -1
ACRBP     1
ACSL6     1
ACTN1     -1
ACTR3     1
ACVR1     1
ADAM10    1
ADAM15    -1
ADAM22    1
ADAMDEC1          1
ADAMTS13          -1
ADAMTS6 1
ADARB1    1
ADD1      -1
ADIPOR1 1
ADNP      1
ADPRHL2 -1
AFAP1     1
AGER      -1
AGK       1
```

Figure S1. Sample input file of deregulated TFs/genes. (1), and (-1) refer to up-and down regulation, respectively.

mirna.bc.diff.txt

```
hsa-let-7a-2      -1
hsa-let-7a-3      -1
hsa-let-7b        -1
hsa-let-7c        1
hsa-let-7d        -1
hsa-let-7f-1      -1
hsa-let-7f-2      -1
hsa-let-7i        1
hsa-mir-100       1
hsa-mir-101-1     -1
hsa-mir-103-1     -1
hsa-mir-106b      1
hsa-mir-10a       1
hsa-mir-10b       -1
hsa-mir-122       1
hsa-mir-124-2     1
hsa-mir-124-3     1
hsa-mir-125a      -1
hsa-mir-125b-1    1
hsa-mir-126       -1
hsa-mir-1264      -1
hsa-mir-1267      -1
hsa-mir-127       1
hsa-mir-1274b     1
hsa-mir-1284      1
hsa-mir-1290      1
hsa-mir-1323      1
hsa-mir-133a-1    -1
```

Figure S2. Sample input file of deregulated miRNAs. (1), and (-1) refer to up-and down regulation, respectively.

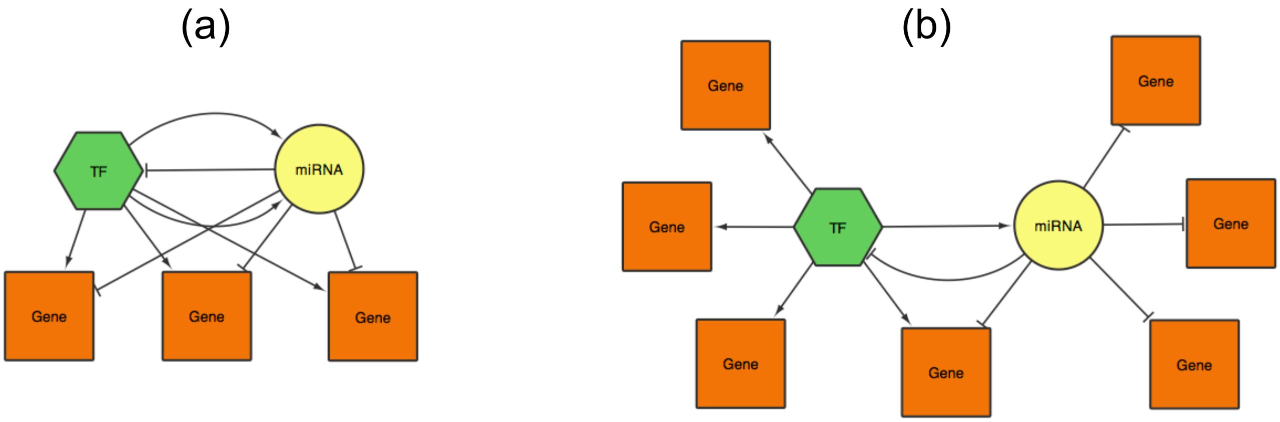

Figure S3. (a) Co-targeted genes defined as genes that are targeted by the same TF and miRNA pair. (b) Co-regulated genes defined as all genes regulated by the TF and the miRNA of this TF-miRNA pair.

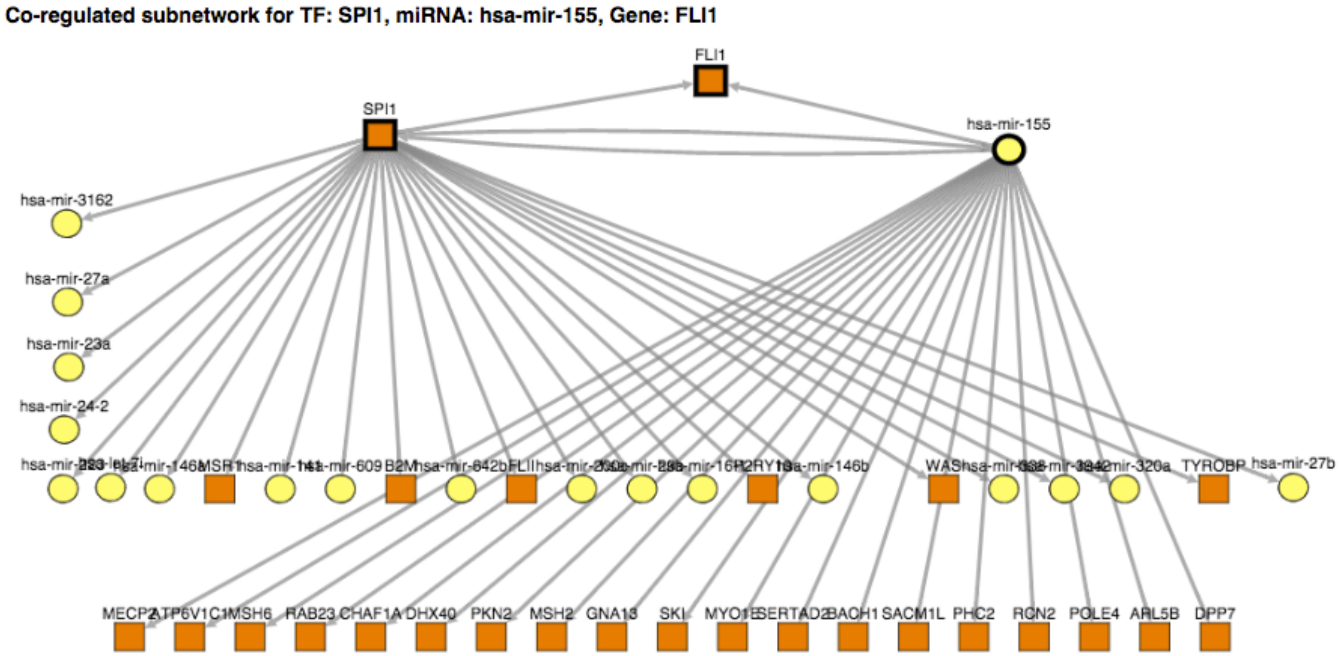

Figure S4. A composite FFL motif involves the TF *SPI1*, the miRNA *has-mir-155*, and the target gene *FLI1*. The co-regulated nodes are also visualized and are further tested whether they compose a cooperative functional module in breast cancerogenesis (see Fig S5).

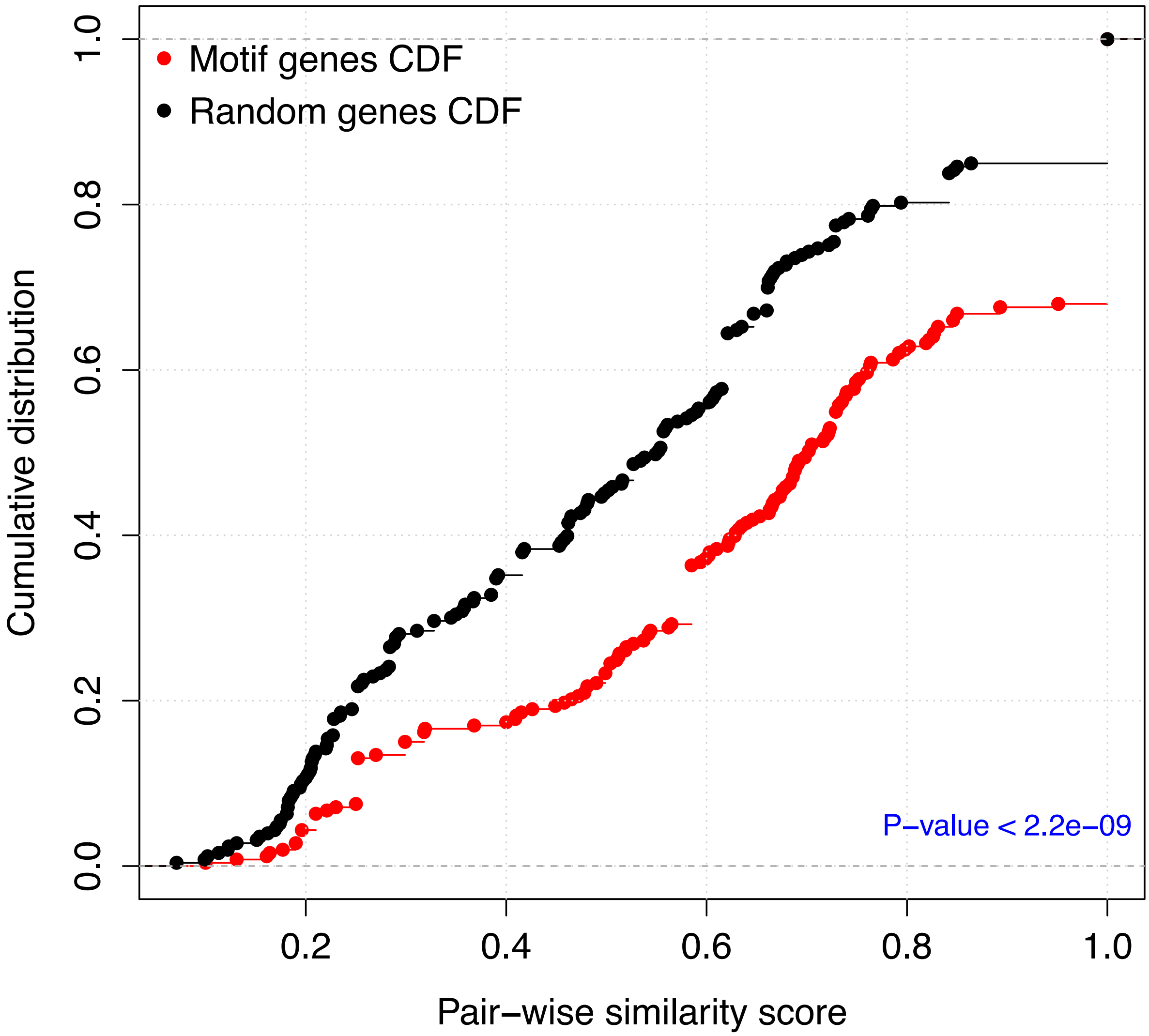

Figure S5. Cumulative distribution of GO functional semantic scores of gene pairs of co-regulated genes in the examined motif (red) versus randomly selected genes (black). The p-value was calculated using the Kolmogorov-Smirnov test.

REFERENCES

1. Matys, V., Fricke, E., Geffers, R., Gößling, E., Haubrock, M., Hehl, R., Hornischer, K., Karas, D., Kel, A.E. and Kel-Margoulis, O.V. (2003) TRANSFAC®: transcriptional regulation, from patterns to profiles. *Nucleic acids research*, **31**, 374-378.
2. Griffith, O.L., Montgomery, S.B., Bernier, B., Chu, B., Kasaian, K., Aerts, S., Mahony, S., Sleumer, M.C., Bilenky, M. and Haeussler, M. (2008) ORegAnno: an open-access community-driven resource for regulatory annotation. *Nucleic acids research*, **36**, D107-D113.
3. Jiang, C., Xuan, Z., Zhao, F. and Zhang, M.Q. (2007) TRED: a transcriptional regulatory element database, new entries and other development. *Nucleic acids research*, **35**, D137-D140.
4. Wang, J., Lu, M., Qiu, C. and Cui, Q. (2010) TransmiR: a transcription factor–microRNA regulation database. *Nucleic acids research*, **38**, D119-D122.
5. Qiu, C., Wang, J., Yao, P., Wang, E. and Cui, Q. (2010) microRNA evolution in a human transcription factor and microRNA regulatory network. *BMC systems biology*, **4**, 90.
6. Yang, J.-H., Li, J.-H., Jiang, S., Zhou, H. and Qu, L.-H. (2013) ChIPBase: a database for decoding the transcriptional regulation of long non-coding RNA and microRNA genes from ChIP-Seq data. *Nucleic acids research*, **41**, D177-D187.
7. Hsu, S.-D., Lin, F.-M., Wu, W.-Y., Liang, C., Huang, W.-C., Chan, W.-L., Tsai, W.-T., Chen, G.-Z., Lee, C.-J. and Chiu, C.-M. (2010) miRTarBase: a database curates experimentally validated microRNA–target interactions. *Nucleic acids research*, gkq1107.
8. Sethupathy, P., Corda, B. and Hatzigeorgiou, A.G. (2006) TarBase: A comprehensive database of experimentally supported animal microRNA targets. *Rna*, **12**, 192-197.
9. Xiao, F., Zuo, Z., Cai, G., Kang, S., Gao, X. and Li, T. (2009) miRecords: an integrated resource for microRNA–target interactions. *Nucleic acids research*, **37**, D105-D110.
10. Yang, J.-H., Li, J.-H., Shao, P., Zhou, H., Chen, Y.-Q. and Qu, L.-H. (2011) starBase: a database for exploring microRNA–mRNA interaction maps from Argonaute CLIP-Seq and Degradome-Seq data. *Nucleic acids research*, **39**, D202-D209.
11. Sengupta, D. and Bandyopadhyay, S. (2011) Participation of microRNAs in human interactome: extraction of microRNA–microRNA regulations. *Molecular Biosystems*, **7**, 1966-1973.
12. Hamed, M.,Spaniol, C., Zapp, A. and Helms, V. (2015) Integrative network based approach identifies key genetic elements in breast invasive carcinoma. BMC Genomics (accepted for publication).
